# Evaluating Counseling for Choice in Malawi: A Client-Centered Approach to Contraceptive Counseling

**DOI:** 10.9745/GHSP-D-22-00319

**Published:** 2023-04-28

**Authors:** Amanda Kalamar, Kendal Danna, Alexandra Angel, Claire W. Rothschild, Innocent Meja, Eva Lathrop, Philip Mkandawire

**Affiliations:** a Population Services International, Washington, DC, USA.; b Population Services International/Malawi, Lilongwe, Malawi.

## Abstract

**Introduction:**

High-quality contraceptive counseling is critical for supporting full, free, and informed contraceptive decision-making. However, the quality of family planning counseling remains poor globally and is too often not tailored to the individual client. The Counseling for Choice (C4C) approach comprises provider tools and training to structure counseling to center clients’ self-identified priorities and to provide relevant information and anticipatory side effects counseling.

**Methods:**

Providers at 25 public and 20 private facilities in Malawi were trained in the C4C approach. Between October and December 2018, we enrolled women seeking contraceptive services in intervention facilities and in matched comparison clinics in a quasi-experimental study. We collected data immediately before and after contraceptive services were received. We used multilevel logistic regression to compare dimensions of women’s counseling experience.

**Results:**

Of 1,179 participants, women counseled by C4C-trained providers rated their providers higher on several quality dimensions, including enabling informed decision-making (11.1% of the comparison group rated their provider as excellent versus 34.4% in intervention), respectful care (35.0% comparison versus 51.3% intervention), and information given about side effects (38.1% comparison versus 72.5% intervention).

**Conclusion:**

In Malawi, C4C improved the quality of care that clients received and their client experience relative to standard counseling. Counseling approaches that center clients’ priorities and provide enhanced anticipatory side effects counseling show promise in improving contraceptive counseling experiences and the quality of care that clients receive.

## Abstract (precis)

Authors found that clients counseled using the Counseling for Choice approach in Malawi reported better quality of contraceptive counseling and experience of care using metrics including person-centered care and quality of information exchange.

## Teaser

**Key Findings:**

- Counseling for Choice (C4C) addresses the interpersonal relations domain of quality of care by anchoring the counseling approach in the core elements of respect, dignity, and empathy and in care that is nondiscriminatory and responsive to unique client needs.
- Women counseled by providers trained in the C4C approach rated their experience more positively against all 4 person-centered contraceptive counseling measures.
- Women counseled by C4C-trained providers rated their providers higher on several quality dimensions, including provision of information to make an informed decision, respectful care, and counseling on side effects.

**Key Implication:**

- Client-centered counseling approaches that structure information provision and interpersonal care based on clients’ priorities and that provide enhanced anticipatory side effects counseling are a promising strategy for improving the quality of counseling and supporting informed and autonomous contraceptive decision-making globally.

## INTRODUCTION

A human rights–based approach to family planning (FP) programming addresses all levels of the health care system and the surrounding enabling environment to ensure the autonomy, agency, and satisfaction of FP clients.^1^ Access to high-quality information and counseling—in addition to affordable, voluntary, and nondiscriminatory contraceptive services and products—is a critical “lever” to pull to achieve quality of care within this system.^1^^,^^2^ Information exchange and interpersonal relations that occur between FP providers and clients have long been recognized as fundamental aspects of quality of care.^3^^,^^4^ Beyond the objective to uphold the client’s right to receive high-quality services, FP clients’ perceptions of quality have also been found to be associated with better contraceptive use dynamics, including increased voluntary method uptake, method satisfaction, and continuation in some settings, although evidence is mixed.^5^^–^^7^ Similarly, anticipatory side effects counseling—counseling that prepares women for contraceptive-induced bleeding changes and other side effects linked with using specific methods that they may experience—has been shown to increase method satisfaction and decrease discontinuation.^8^^,^^9^ However, evidence on structured counseling approaches that improve the quality of information sharing and interpersonal relations, as well as women’s experiences using contraception, remains weak.^10^

As a result, the quality of FP counseling remains poor globally. A recent analysis of Demographic and Health Survey data from 25 low- and middle-income countries found that the average country-level Method Information Index score was 34%—meaning that only one-third of current contraceptive users received counseling on more than 1 method, were told about side effects, and were told what to do if side effects occurred.^11^ Despite overwhelming evidence that fear and experience of adverse side effects and health concerns are major drivers of contraceptive nonuse and method-related discontinuation among women who wish to avoid pregnancy,^12^^–^^13^ counseling approaches widely used by FP providers in low- and middle-income countries lack an adequate focus on anticipatory side effects counseling.^14^ Evidence-based approaches that focus on improving care across these domains—approaches that are tailored to the client’s unique needs, improve information sharing and the client-provider relationship, and strengthen anticipatory side effects counseling—are urgently needed to support informed method choice aligned with clients’ preferences and to reduce negative contraceptive use experiences.

Counseling for Choice (C4C) is a new FP counseling approach developed by Population Services International, publicly available at https://www.psi.org/C4C.^15^ C4C, which comprises a provider training curriculum and job aid, replaces traditional tiered-effectiveness counseling with structured counseling based on the method attributes most valued by the individual client. C4C also provides a guided structure for comprehensive anticipatory side effects counseling, with a particular focus on menstrual bleeding changes. We used a quasi-experimental study design to evaluate the impact of the C4C intervention on the quality of counseling received, measured by clients’ experiences.

C4C replaces traditional tiered-effectiveness counseling with structured counseling based on the method attributes most valued by the individual client.

## C4C APPROACH FOR FP COUNSELING

Contraceptive counseling has evolved as contraceptive approaches and tools have been iteratively developed and updated to improve quality of care. To counsel patients thoroughly on their choices, many clinicians use the autonomous approach to counseling. This involves providing information on all available, medically appropriate methods, with the patient subsequently deciding on a method with minimal provider input.^16^ Another common approach is the tiered-effectiveness method. With an effectiveness framework, clinicians present the most effective options first—highlighting voluntary long-acting reversible contraceptive methods.^17^ One approach that bridges the gap between the directive versus autonomous approaches is the shared decision-making model—a method that recognizes the expertise of both the provider, who has comprehensive information about methods from a clinical perspective, and the clients, who best understand their own needs and preferences.^18^ This and other common counseling approaches, such as Balanced Counseling Strategy Plus (BCS+), employ evidence-based best practices shown to improve quality of care and FP outcomes, such as increased uptake.^19^ These tools are widely used in FP programs globally; however, research on the effectiveness of specific approaches and tools to improve person-centered care and impact contraceptive use dynamics is limited.^20^

Among available counseling tools, the new C4C approach shares some common components with BCS+, the contraceptive counseling tool developed by Population Council and used across many low- and middle-income countries.^21^ BCS+ also prioritizes the demedicalization of provider language during counseling, uses client-centered and shared decision-making approaches, and incorporates specific job aids.^22^ From there, BCS+ and C4C diverge. BCS+ integrates tiered-effectiveness counseling into the approach, while C4C recognizes that clients may place a higher value on alternative method benefits—such as use on-demand, low frequency of provider visits required, or immediate return to fertility—and makes it easy for providers to compare contraceptives in relation to these other benefits. Where the algorithm, cards, and medical eligibility criteria information used by BCS+ are separate tools, C4C integrates the full suite of information and tools into a single, all-encompassing job aid. Different than BCS+ cards, the C4C job aid includes pages specifically meant to be viewed by lower-literacy clients. Finally, recognizing that experiencing side effects is a frequently cited reason for method discontinuation,^12^ C4C places a focus on anticipatory side effects counseling. While this comparison between C4C and BCS+ is meant to provide a well-known reference point for the community of practice familiar with this tool, our research does not seek to compare these 2 approaches.

### C4C Intervention and Tools

Foundational to the C4C approach are 3 contraceptive counseling tenets: support the client to make an informed decision through clear and relevant information provision; provide high-quality, client-centered interpersonal care; and create a dialogue with clients about side effects, including what to expect and how to manage them.

Three tenets inform C4C’s approach: support the client to make an informed decision through clear and relevant information provision; provide high-quality, client-centered interpersonal care; and create a dialogue with clients about side effects.

**FIGURE 1:**
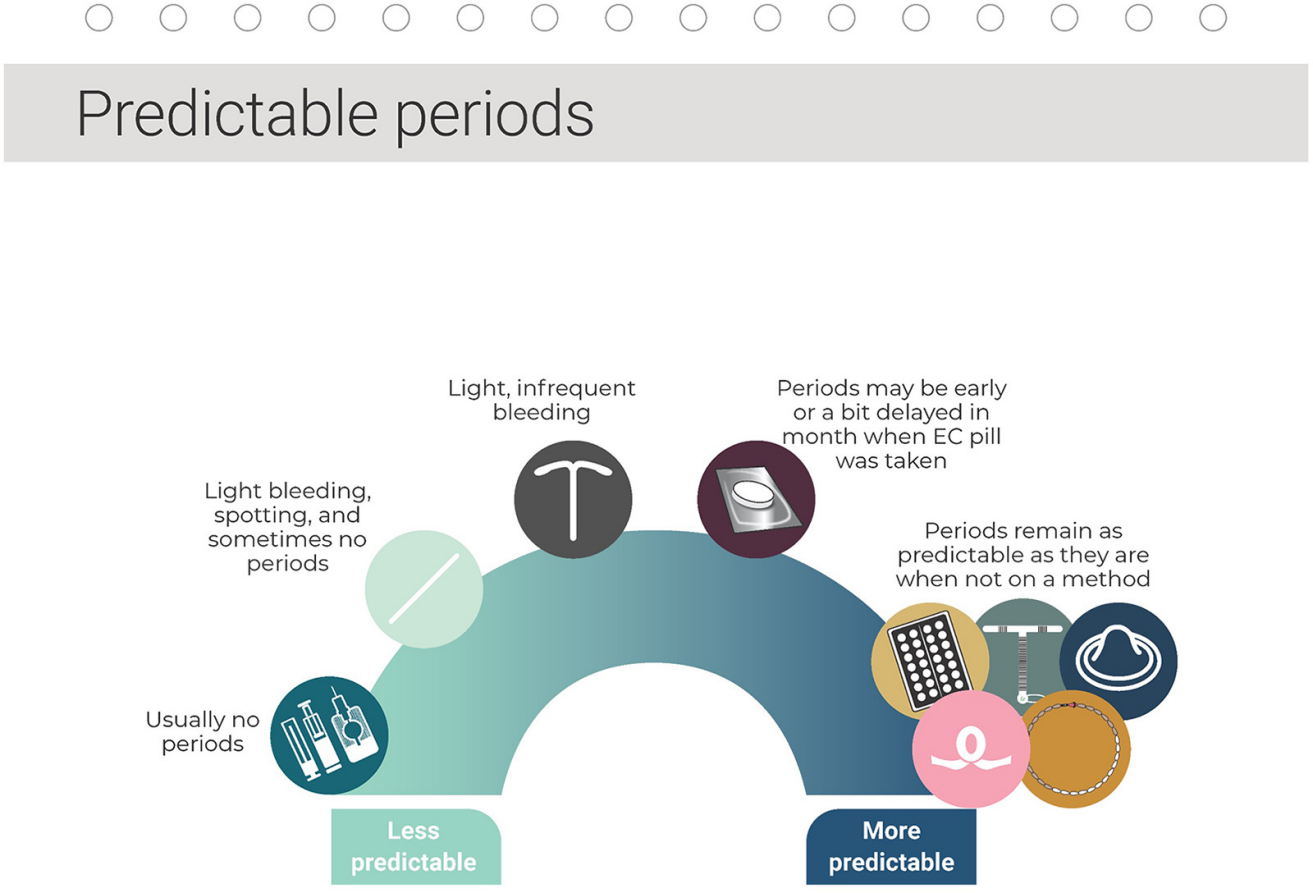
Example of Counseling on Specific Method Attributes From the C4C Choice Book Job Aid Abbreviation: C4C, Counseling For Choice; EC, emergency contraception.

The C4C approach has 2 key components: a 3-day training for providers and the Choice Book job aid for providers to use during counseling (Box). The training provides multiple tools and tech-niques to improve the counseling interaction by creating a dialogue about what matters to the client rather than using the counseling session as a didactic or rote lecture to impart the provider’s perspective (and potential bias) and a long list of facts. The Choice Book is a job aid for providers that includes both provider-facing and client-facing tools, including existing reference tools from the World Health Organization and other sources. Figure 1 shows an example book page illustrating how methods are compared across different attributes.

BOX Components of the Counseling for Choice Approach

Provider training:

- 3 days
- Training modules and activities reviewing Counseling for Choice (C4C) counseling principles
- Role-play with the Choice Book

Choice Book (C4C provider job aid):

- Counseling matrix: a tool illustrating which contraceptive options offer various contraceptive and lifestyle benefits
- GATHER: demonstrating how C4C aligns with the GATHER (“Greet, Ask, Help, Explain, and Return”) approach^23^
- Benefit-specific pages: comparing each method option relative to whether it offers a particular benefit (Figure 1)
- Method-specific pages: in-depth information about each method, including the 3 Ws: what to do, what to expect, when to come back
- Other resources and reference tools:
  - NORMAL tool for counseling on contraceptive induced menstrual bleeding changes^24^
  - Quick-start reference for breastfeeding and postabortion clients
  - World Health Organization Medical Eligibility Criteria
  - Job aid for DMPA reinjection
  - DMPA-SC self-injection instructions
  - Job aid for ruling out pregnancy
  - Instructions for management of side effects
  - Scale image of uterus

## METHODS

### Study Design

We conducted a quasi-experimental evaluation with an intervention and concurrent comparison group in 50 public and 40 private facilities in 8 districts in Northern, Central, and Southern Malawi (Dwanga, Lilongwe, Mangochi, Mchinji, Mzuzu, Nkhata Bay, Nsanje, and Salima). Intervention facilities were sampled through stratified random sampling of a full roster of facilities offering FP services and counseling. Facilities were stratified first by district, then by public or private sector, and finally by client load. Our goal was, within the districts, to balance the number of public and private facilities with high, medium, and low client flows in the intervention and control groups. Of 30 public and 30 private facilities sampled, 25 and 20, respectively, consented to participate in the C4C intervention. We then selected matched comparison facilities based on FP client volumes and sector (private or public). Included facilities were primarily FP and reproductive health clinics, including franchises, and hospitals with FP and reproductive health services or wards. During the study period, providers in the comparison group continued using tools with which they were well versed and familiar, such as the flipchart approved by the Ministry of Health; the comparison group was not instructed to use a specific FP counseling tool or approach. Selected providers in intervention facilities received a 3-day training on the C4C approach using the Choice Book that would guide the counseling experience. This training included role-play and practice to achieve competency in the counseling approach, which was assessed via quizzes and observation by the lead trainer. Half of the providers who participated in the training were nurse midwife technicians, about one-quarter were medical assistants, and the remaining one-quarter were either clinical officers or nurse midwife assistants. A post hoc review of trainings that all providers in both groups had received in the past 3 years revealed little difference between comparison (standard-of-care) and intervention providers in terms of training received before the C4C intervention.

### Study Population

Between October and December 2018, we enrolled clients seeking FP services at intervention or comparison facilities. All women of reproductive age (aged 18–49 years) seeking FP services—including those initiating contraception, switching methods, or continuing method use—were eligible to participate in in-person study procedures on the date of enrollment. No compensation was provided for participation in the study.

### Data Collection

Data collection began 3 months after the training to allow providers time to become accustomed to the C4C approach. Participants completed 2 surveys on the date of enrollment: a pre-counseling survey before seeing a provider and a second post-counseling survey immediately after seeing a provider. Both the pre- and post-counseling surveys were administered in person in a private area of the clinic. The pre-counseling survey captured demographic information, contraceptive history, and acceptability of specific contraceptive side effects. The post-counseling survey collected information on the method chosen and reasons for selection (including reasons for selecting no method), content of information received during the counseling session, and satisfaction with the counseling experience. Participants were asked in the post-counseling survey to identify their provider; in the final analysis sample, participants who visited an intervention facility but who received counseling from a provider not trained in C4C were excluded.

### Ascertainment of Dependent Variables

We ascertained perceived quality of care using the validated 4-item Person-Centered Contraceptive Counseling scale,^25^ which includes individual items on clients’ perceptions of the respectfulness of care, whether they were allowed to voice their contraceptive method preferences, whether they felt their preferences were taken seriously, and whether they felt that they received adequate information to make a decision about a contraceptive method. Individual items are measured on a 5-point Likert scale (poor, fair, good, very good, or excellent). We report the items that comprise the Person-Centered Contraceptive Counseling scale individually and as a summative binary variable, equal to 1 if the highest rating (“excellent”) was given for all 4 items and 0 if otherwise according to published scale scoring guidance.^26^

Additional nonvalidated measures were developed to measure key C4C quality domains. For example, within the domain of information exchange and interpersonal relations, confidence using the chosen method was measured on a 5-point Likert scale (from “not at all” to “very confident”); in addition, binary (yes/no) variables were captured on whether the provider addressed all concerns about using contraception, whether the provider asked about prior contraceptive experience, whether the participant trusted the provider to keep the consultation private, and whether the provider helped make a plan for how to remember to use the method (among participants who selected to use short-term methods). In the side effects expectations and management domain, we captured 3 binary (yes/no) variables: whether the provider provided information on potential side effects, whether the provider helped plan to manage potential side effects, and whether the participant anticipated discontinuing her method immediately if she experienced side effects. A table in the Supplement provides further detail on how all independent variables are linked to each of our 3 quality of care domains of interest.

### Statistical Analysis

To estimate the effect of C4C on quality received, we compared participants at intervention versus comparison facilities by fitting multilevel mixed effects models with robust standard errors, with individuals nested within facilities. For Likert scale outcomes, we fit multilevel logistic regression models with random intercepts for health facilities to estimate odds ratios (OR), which can be interpreted as the odds that women in the intervention group gave the highest rating on the Likert scale compared to women in the comparison group. For binary outcome variables, we used analogous mixed effects logistic regression models. Adjusted models include covariates for age (specified as a continuous variable), marital status (modeled categorically as currently married, living with a man as if married, or not currently married or living with a male partner), highest level of educational attainment (none, primary, secondary, or higher), number of living children (none, 1–2, 3–4, or 5 or more), contraceptive method type received at consultation (including none, if no method was chosen after counseling), and facility sector (public or private). The analysis was conducted using STATA version 15.1.

### Ethical Approval

The study was approved by the Research Ethics Board of Population Services International in Washington, DC, and by the National Committee on Research in the Social Sciences and Humanities in Malawi. The district health management team and the head/owner of each participating facility gave permission for data collection at study sites. All participants gave their verbal informed consent before study procedures. The clients in both intervention and control sites gave their consent to participate in the study. The study participants in both intervention and control sites were briefed on the study objectives and all requirements of the consenting process.

## RESULTS

A total of 1,179 women were enrolled for the in-person study components (N=578 in the comparison group and N=601 in the intervention group). In the full baseline sample, participants were evenly distributed across age groups, with a slightly higher proportion of women aged 18–24 years and a slightly lower proportion of women aged 35 years and older (Table 1). Most women (520 [90%]) were married and had 1 or more children. In the intervention group, 391 women (69%) chose injectable contraception and 121 (21%) chose implants, while in the comparison group, injectable contraception was more common, and implants were less so (401 [85%] and 31 [7%], respectively).

**TABLE 1.** Demographics of Counseling for Choice Evaluation Participants in Malawi

	**Control, No. (%) (N=578)**	**Intervention, No. (%) (N=601)**	***P* Value**
Age, years			.73
18–24	208 (36.0)	228 (37.9)	
25–29	146 (25.3)	155 (25.8)	
30–34	115 (19.9)	119 (19.8)	
35+	109 (18.9)	99 (16.5)	
Marital status			.95
Married or living together	520 (90.0)	540 (89.9)	
Not married	58 (10.0)	61 (10.2)	
Children, no.			.74
0	5 (0.9)	5 (0.8)	
1	161 (27.9)	147 (24.5)	
2	153 (26.5)	165 (27.5)	
3	104 (18.0)	119 (19.8)	
4+	155 (26.8)	165 (27.5)	
Method use before visit			.41
Not currently using	99 (17.1)	114 (19.0)	
Currently using	479 (82.9)	487 (81.0)	
Method chosen at baseline			<.001
None	105 (18.2)	33 (5.5)	
Female sterilization	0 (0)	2 (0.4)	
IUD	2 (0.4)	2 (0.4)	
Implant	31 (6.6)	121 (21.3)	
Injectable	401 (84.8)	391 (68.8)	
Oral contraceptive pills	28 (5.9)	43 (7.6)	
Condoms only	8 (1.7)	8 (1.4)	
Emergency contraceptive pills	3 (0.6)	1 (0.2)	
Facility type			.62
Public sector	265 (45.8)	267 (44.4)	
Private sector	313 (54.2)	334 (55.6)	

Abbreviation: IUD, intrauterine device.

### Client Satisfaction and Experience of Quality of Care

**FIGURE 2:**
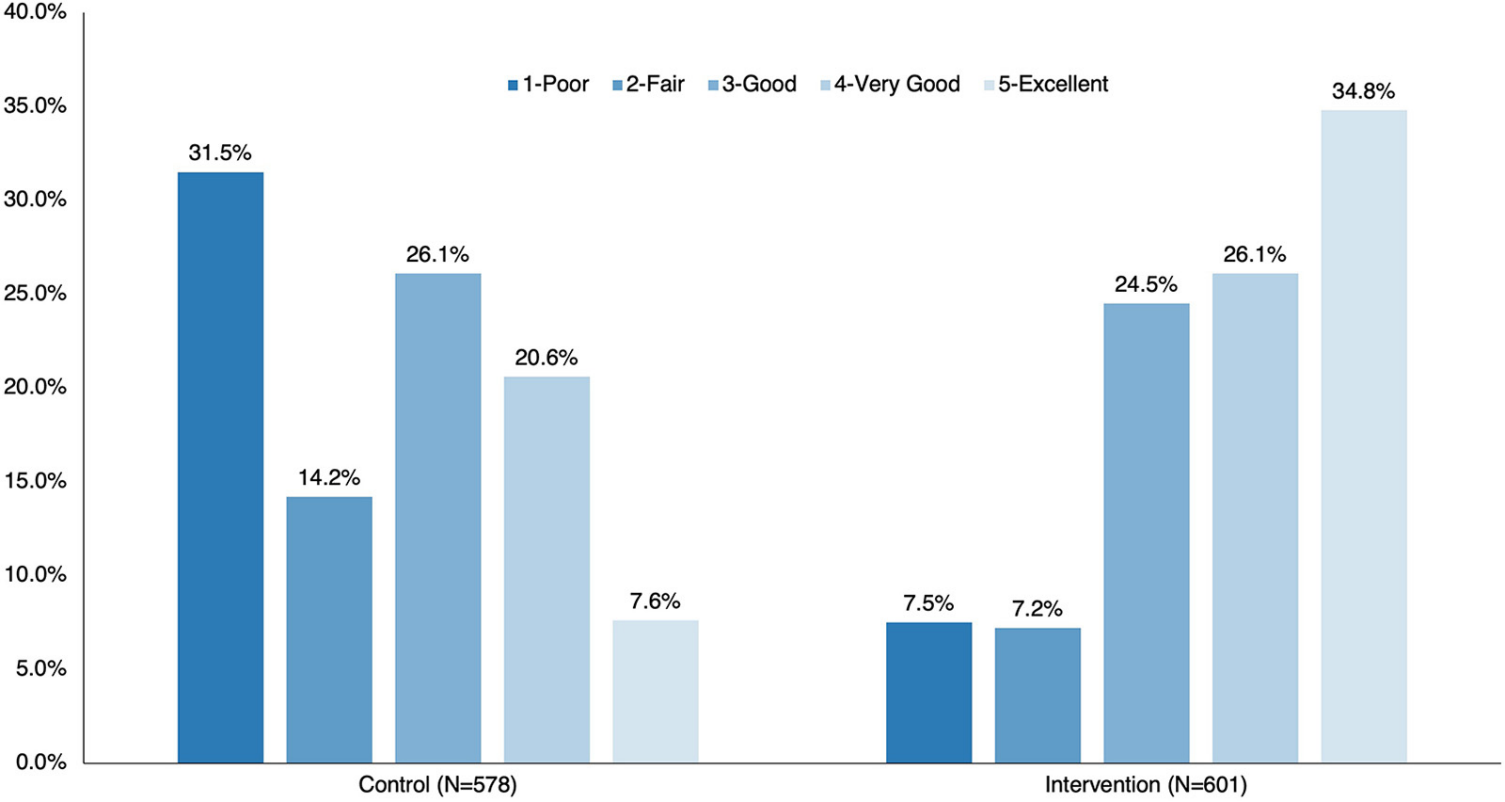
Client Rating of Overall Counseling Experience Immediately Post-Counseling

More women rated their overall counseling experience as poor in the comparison group (32%) compared to the intervention group (8%), while more women in the intervention group rated their experience as excellent (35%) compared to women in the comparison group (8%) (Figure 2).

More women in the intervention group rated their experience as excellent compared to women in the comparison group.

Receipt of care from C4C-trained providers was associated with statistically significant, positive odds of rating the provider as “excellent” (the highest score) on 4 questions (Table 2): respecting you as a person (adjusted odds ratio [aOR]=2.65; 95% confidence interval [CI]=1.40, 5.02); letting you say what matters to you about your contraceptive method (aOR=2.20; 95% CI=1.20, 4.00); taking your preferences about contraception seriously (aOR=2.60; 95% CI=1.42, 4.75); and giving enough information to make the best decision about a method (aOR=5.14; 95% CI=2.72, 9.71). Participants in the intervention group had 4.6 times the odds of rating their provider as “excellent” on all 4 questions as the comparison group: 140 participants (23.3%) in the intervention group rated their provider as “excellent” on all 4 questions, relative to just 36 (6.2%) in the comparison group.

**TABLE 2.** Person-Centered Contraceptive Counseling: Post-Counseling Results Among Women in the Counseling for Choice Evaluation, Malawi

**How do you think the provider did in:**	**Control, No. (%)**	**Intervention, No. (%)**	**OR**^a^ **(95% CI)**	**aOR**^b^ **(95% CI)**	**ICC** ^c^	**σ_u_^2^** ^d^
Respecting you as a person?						
1 - Poor	18 (3.1)	9 (1.5)	2.62^e^ (1.38, 4.97)	2.65^e^ (1.40, 5.02)	0.37	1.71
2 - Fair	60 (10.4)	18 (3.0)				
3 - Good	153 (26.5)	118 (19.6)				
4 - Very good	145 (25.1)	148 (24.6)				
5 - Excellent	202 (35.0)	308 (51.3)				
Letting you say what matters to you about your contraceptive method?						
1 - Poor	88 (15.2)	37 (6.2)	2.37^e^ (1.34, 4.20)	2.20^f^ (1.20, 4.00)	0.32	1.40
2 - Fair	81 (14.0)	32 (5.3)				
3 - Good	147 (25.4)	140 (23.9)				
4 - Very good	103 (17.8)	148 (24.6)				
5 - Excellent	159 (27.5)	244 (40.6)				
Taking your preferences about contraception seriously?						
1 - Poor	50 (9.0)	20 (3.3)	2.97^e^ (1.64, 5.37)	2.60^e^ (1.42, 4.75)	0.34	1.43
2 - Fair	102 (17.7)	36 (6.0)				
3 - Good	145 (25.1)	143 (23.8)				
4 - Very good	124 (21.5)	152 (25.3)				
5 - Excellent	155 (26.8)	250 (41.6)				
Gave enough information to make the best decision about a contraceptive method						
1 - Poor	223 (38.6)	75 (12.5)	5.68^e^ (3.01, 10.73)	5.14^e^ (2.72, 9.71)	0.43	1.63
2 - Fair	103 (17.8)	56 (9.3)				
3 - Good	132 (22.8)	138 (23.0)				
4 - Very good	56 (9.7)	125 (20.8)				
5 - Excellent	64 (11.1)	207 (34.4)				
PCCC Measure						
At least one item with less than highest score (“5”)	542 (94.8)	461 (76.7)	4.76^e^ (1.92, 11.81)	4.59^e^ (1.86, 11.33)	0.55	2.95
Highest score (“5”) on all items	36 (6.2)	140 (23.3)				

Abbreviations: aOR, adjusted odds ratio; CI, confidence interval; ICC, intraclass correlation; OR, odds ratio; PCCC, person-centered contraceptive counseling.

^a^ Estimated from multilevel mixed effects model with ordinal or binary outcomes. Unadjusted OR estimated from bivariate models.

^b^ Estimated from models that include demographic variables (age, marital status, and education), a categorical variable for number of living children (defined as none, 1–2, 3–4, or 5 or more), and variables for method chosen at provider visit (including no method chosen) and type of facility (public or private). All adjusted models comprised of 1,179 observations.

^c^ Estimated from unadjusted model with random intercepts for health facility but without fixed effects predictors.

^d^ Estimated variance for the random effects at the health facility level; estimate has not been exponentiated.

^e^ Significant at *P*<.01.

^f^ Significant at *P*<.05.

The person-centered contraceptive counseling measures described in Table 2 are related to aspects of both domains of information exchange and interpersonal relations. In addition to these validated measures, we measured other aspects of counseling related to these domains with the additional variables in Table 3. Participants had 6-fold odds (aOR=6.4; 95% CI=3.08, 13.4) of rating their provider as excellent in addressing all concerns about their contraceptive method relative to those in the comparison group (Table 3). They were also more likely to report that their provider asked about their previous contraceptive experiences than clients in the comparison group, with 448 (74.5%) reporting “yes” versus 219 (37.9%) in the comparison (OR=6.76; 95% CI=3, 12.92). Clients choosing short-acting methods in the intervention group were more likely to report being helped to make a plan to use their method correctly (453 [79.8%] versus 258 [54.6%], respectively; aOR=6.45; 95% CI=2.57, 16.2). Clients in the intervention group were also more likely to report that they trusted their provider to keep their discussion confidential (575 [96.7%] versus 501 [86.7%]; (aOR=3.06; 95% CI=1.4, 6.67). Lastly, participants were more likely to rate that they were “very confident” in their choice of method in the intervention (280 [49.6%]) versus the comparison group (176 [37.5%]) (OR=1.94, 95% CI=1.0, 3.4), although this difference was not significant at the *P*=.05 level (*P*=.057).

**TABLE 3.** Informed Decision-Making and Interpersonal Communication: Post-Counseling Results Among Women in the Counseling for Choice Evaluation, Malawi

	**Control, No. (%)**	**Intervention, No. (%)**	**OR**^a^ **(95% CI)**	**aOR**^b^ **(95% CI)**	**ICC** ^c^	**σ_u_^2^** ^d^	**Observations** ^e^
Provider addressed all concerns about using contraception							
Yes	229 (39.6)	460 (76.5)	7.26^f^ (3.57, 14.80)	6.43^f^ (3.08, 13.41)	0.48	1.85	1,176
No	348 (60.2)	140 (23.3)					
							
Provider asked about past contraceptive experience							
Yes	219 (37.9)	448 (74.5)	6.76^g^ (3.35, 13.65)	6.23^f^ (3.00, 12.92)	0.45	1.84	1,176
No	359 (62.1)	153 (25.5)					
							
Trust provider will keep information discussed during consultation a secret							
Yes	501 (86.7)	575 (96.7)	3.23^f^ (1.50, 6.97)	3.06^f^ (1.41, 6.67)	0.34	1.14	1,163
No	27 (4.7)	8 (1.3)					
Don't know^h^	50 (8.7)	18 (3.0)					
							
Provider helped make a plan for how to remember to use the method, among short-term method users^i^							
Yes	258 (54.6)	453 (79.8)	6.32^f^ (2.60, 15.35)	6.45^f^ (2.57, 16.20)	0.49	2.58	1,034
No	215 (45.5)	115 (20.3)					
							
Confidence using chosen method, among those who received a method during the consultation							
1 - Not at all	25 (5.3)	10 (1.8)	1.94^g^ (1.04, 3.62)	1.84 (0.99, 3.44)	0.33	1.49	1,035
2 - No opinion	27 (5.7)	23 (4.1)					
3 - Somehow confident	104 (22.1)	63 (11.2)					
4 - Confident	138 (29.4)	189 (33.5)					
5 - Very confident	176 (37.5)	280 (49.6)					

Abbreviations: aOR, adjusted odds ratio; CI, confidence interval; ICC, intraclass correlation; OR, odds ratio.

^a^ Estimated from multilevel mixed effects models including facility-level random effects. “Gave enough information” and “confidence to use chosen method” were modeled as ordinal variables. The “provider addressed all concerns” outcome was modeled as a binary variable. Odds ratios estimated from multilevel mixed effects model with ordinal or binary outcomes. Unadjusted odds ratios estimated from bivariate models.

^b^ Estimated from models that include demographic variables (age, marital status, and education), a categorical variable for number of living children (defined as none, 1–2, 3–4, or 5 or more), and variables for method chosen at provider visit and type of facility (public or private).

^c^ Estimated from unadjusted model with random intercepts for health facility but without fixed effects predictors.

^d^ Estimated variance for the random effects at the health facility level in the adjusted model; estimate has not been exponentiated.

^e^ Number of observations in the adjusted model; complete case analysis.

^f^ Significant at *P*<.01.

^g^ Significant at *P*<.05.

^h^ For modeled estimates, we use a binary version of the indicator that combines "no" and "don't know" responses in a single category (versus “yes”).

^i^ Asked only of women who received injectables, oral contraceptive pills, condoms, or emergency contraceptive pills.

### Side Effects Expectations and Management

Women in the intervention group were more likely to report that the provider told them about possible side effects they might experience (412 [73%]) versus the comparison group (180 [38%]) (aOR=5.98; 95% CI=2.97, 12.03) (Table 4). Intervention group participants were also significantly more likely to report that their provider had helped them make a plan to manage side effects (441 [78%] versus 194 [41%] in the comparison group; aOR=8.79; 95% CI=3.68, 21.01). Fewer women in the intervention group reported that they would discontinue their method immediately if they experienced side effects (40 [7%]) versus the comparison group (52 [11%]), although this difference was not statistically significant.

**TABLE 4.** Side Effects Expectations and Management: Baseline Results Among Women in Control and Intervention Groups, Among Women Who Received a Method

	**Did the Provider Tell You About Side Effects You Might Experience With Your Chosen Method?**		** **	**Did the Provider Help You Make a Plan to Manage Side Effects?**		** **	**If You Experience Side Effects, Will You Discontinue Your Method Immediately?**	
	**Control, No. (%)**	**Intervention, No. (%)**	** **	**Control, No. (%)**	**Intervention, No. (%)**	** **	**Control, No. (%)**	**Intervention, No. (%)**
Yes	180 (38.1)	412 (72.5)		194 (41.0)	441 (77.6)		52 (11.0)	40 (7.0)
No	293 (62.0)	156 (27.5)		279 (59.0)	127 (22.4)		421 (89.0)	528 (93.0)
OR^a^ (95% CI)	6.62^b^ (3.24, 13.53)			8.89^b^ (3.77, 20.96)			0.67 (0.40, 1.13)	
aOR^c^ (95% CI)	5.98^b^ (2.97, 12.03)			8.79^b^ (3.68, 21.01)			0.79 (0.45, 1.40)	
ICC^d^	0.45			0.55			0.09	
σ_u_^2e^	1.56			2.59			0.26	
Observations^f^	1,038			1,038			1,024	

Abbreviations: aOR, adjusted odds ratio; CI, confidence interval; ICC, intraclass correlation; OR, odds ratio.

^a^ Estimated from multilevel mixed effects model with ordinal or binary outcomes. Unadjusted odds ratios estimated from bivariate models.

^b^ Significant at *P*<.01.

^c^ Estimated from models that include demographic variables (age, marital status, education), a categorical variable for number of living children (defined as none, 1–2, 3–4, or 5 or more), and variables for method chosen at provider visit and type of facility (public or private).

^d^ Estimated from unadjusted model with random intercepts for health facility but without fixed effects predictors.

^e^ Estimated variance for the random effects at the health facility level; estimate has not been exponentiated.

^f^ Number of observations in the adjusted model.

## DISCUSSION

The novel C4C approach to FP counseling was specifically designed to address common issues with the quality of contraceptive counseling. The approach aims to support the client to make an informed decision about a method that aligns with their self-identified needs and individual preferences for specific method attributes. Clients counseled by C4C providers were more likely to report better care received, with more than 4 times as many reporting their experience as “excellent” overall. We find that the C4C approach improved clients’ experience of care across multiple domains and measures of person-centered care, including information exchange, interpersonal relations, and anticipatory side effects counseling, relative to standard-of-care counseling provided in public and private participating health facilities in Malawi.

We find that the C4C approach improved clients’ experience of care across multiple domains and measures of person-centered care relative to standard-of-care counseling.

The interpersonal relations quality of care domain in FP is critical to an overall high quality of care experience: a systematic review on the effects of person-centered quality of contraceptive care found that interventions to improve person-centeredness were consistently associated with improved client experience, perceptions of quality, and satisfaction.^27^ C4C addresses this domain of quality by anchoring the counseling approach in the core elements of respect, dignity, and empathy and in care that is nondiscriminatory and responsive to unique client needs. Participants in the intervention group of our study consistently rated their providers more positively across indicators of this client-provider relationship, reporting that their providers respected them as a person, let them say what mattered to them, took their preferences seriously, and were trusted to keep their conversation confidential compared to those in the comparison group.

A principal tenet of the C4C approach is to enable informed decision-making through clear and relevant information provision, building on counseling approaches such as the World Health Organization’s Decision-Making Tool for Family Planning Clients and Providers and the Balanced Counseling Strategy tool.^28^ Participants who received the C4C intervention were more likely to report that they had enough information to select a method that fit their needs and had more confidence in their ability to use their chosen method than participants in the comparison group. Their providers were more likely to ask them about their previous contraceptive use and to address all of their concerns. This exchange of information is critical to ensuring that clients are well informed about contraceptive options that best suit them. It includes having appropriate information to prepare them for side effects they may experience with a chosen method, a factor that is directly correlated to contraceptive use experiences, and method satisfaction over time. Clients counseled by C4C providers were more likely to report receiving this anticipatory side effects counseling and having discussed a plan with their provider for how to manage these side effects. Taken together, the findings from this evaluation suggest that the tailored counseling encouraged by the C4C approach, when compared to the standard of care, enables improved information exchange that helps clients make the best contraceptive choice for them. This is consistent with existing literature that describes improved client experiences when counseling includes clear information tailored to one’s expressed needs and preferences.^29^^,^^30^

Our findings suggest that the C4C approach, when compared to the standard of care, enables improved information exchange that helps clients make the best contraceptive choice for them.

While overall counseling received was significantly higher among women in the intervention group, the finding that even women in the intervention group continue to report some dissatisfaction with their counseling experience (14.7% reporting their experience as “fair” or “poor”) indicates that more can be done to further improve counseling, even when using the C4C approach. This study adds to the growing evidence base on the impact of the quality of counseling on client experience. Several studies have found positive effects of interventions to improve client- or person-centeredness and quality of contraceptive counseling on contraceptive use dynamics, hypothesizing that improved perceptions of interpersonal connection with a provider during counseling, having enough information to make an informed choice, and feeling confident to understand and manage side effects may be associated with method initiation and improved method use experiences.^27^^,^^31^^–^^34^ However, evidence of the impact of counseling on contraceptive use dynamics is mixed.^21^^,^^35^^,^^36^ While we do not look here at the impact of the C4C approach on method use over time, we do observe that women counseled using C4C were less likely to report that they would discontinue their method immediately if they experienced side effects, relative to those counseled using the standard approach. Although the difference was not statistically significant, this finding suggests that the C4C approach may support women to select methods with side effect profiles that are more tolerable for their preferences or to better prepare women for what they may expect in terms of side effects. Exploring the impact of improved quality in counseling on contraceptive use dynamics and satisfaction with FP methods over time should be a priority for those in the field aiming to develop and use counseling approaches that truly meet client needs.

### Strengths and Limitations

A primary strength of this study is its inclusion of a robust comparison group that allows for direct comparison of key areas of the counseling experience between women who were counseled by C4C-trained providers and women who were not, allowing for more direct conclusions to be drawn regarding the effect that the C4C approach may have on women’s experiences with a provider.

There are also some limitations. Though unaware of the specific survey questions to clients or which clients would be surveyed, providers in the intervention group were aware that the new C4C approach on which they were trained would be studied, which may have affected adherence levels to the approach. The pre-counseling survey could have acted as an intervention itself or primed respondents to ask their provider about the topics being asked (e.g., about side effects). This may have improved the quality of counseling observed, but the effect would be expected to be nondifferential by treatment group since all participants received the same pre-counseling survey. Lastly, while it was not within the scope of our project to design a separate training for our comparison group, it is possible that improvements in quality of care could have been seen across some of the same indicators studied here regardless of the specific approach used; the act of simply retraining providers in principles of quality counseling could result in better counseling. Further research could explore the comparative impact of the C4C approach against training in other counseling approaches.

## CONCLUSION

This study strengthens the evidence base for the utility and effectiveness of client-centered contraceptive counseling. Among FP clients in Malawi, we found that the C4C approach improved the perceived quality of care across multiple domains relative to standard counseling approaches. Counseling that focuses on supporting clients’ fully informed choice in method selection, improving client-centeredness of the interaction, and strengthening the client’s understanding of the potential side effects of their chosen method is a promising approach to improving contraceptive counseling and use experiences.

## Supplementary Material

GHSP-D-22-00319-supplement.pdf

## Notes

**Peer Reviewed**

**First published online:** April 7, 2023.

## References

[1] FP2030, United Nations Population Fund, What Works Association. The Comprehensive Human Rights-based Voluntary Family Planning Program Framework. FP2030; 2021. Accessed March 8, 2023. https://commitments.fp2030.org/sites/default/files/06.25.21_Framework_Brief.pdf

[2] Senderowicz L. Contraceptive autonomy: conceptions and measurement of a novel family planning indicator. Stud Fam Plann. 2020;51:161–176. 10.1111/sifp.12114. 32358789

[3] Bruce J. Fundamental elements of the quality of care: a simple framework. Stud Fam Plann. 1990;21(2):61–91. 2191476

[4] Jain AK, Hardee K. Revising the FP quality of care framework in the context of rights-based family planning [published correction appears in Stud Fam Plann. 2018 Dec;49(4):397–401]. Stud Fam Plann. 2018;49(2):171–179. 10.1111/sifp.12052. 29708277

[5] Pariani S, Heer D, Van Arsdol MJ. Does choice make a difference to contraceptive use? Evidence from east Java. Stud Fam Plann. 1991;22:384–390. 1792678

[6] Koenig M, Hossain M, Whittaker M. The influence of quality of care upon contraceptive use in rural Bangladesh. Stud Fam Plann. 1997;28:278–289. 9431649

[7] Chakraborty NM, Chang K, Bellows B, et al. Association between the quality of contraceptive counseling and method continuation: findings from a prospective cohort study in social franchise clinics in Pakistan and Uganda. Glob Health Sci Pract. 2019;7(1):87–102. 10.9745/GHSP-D-18-00407. 30846566 PMC6538133

[8] Gilmore K, Ojanen-Goldsmith A, Callegari LS, Godfrey EM. The first 6 months: developing a user-informed anticipatory counselling video about the levonorgestrel intrauterine system. BMJ Sex Reprod Health. 2018;44:248–253. 10.1136/bmjsrh-2018-200055. 29934403

[9] Backman T, Huhtala S, Luoto R, Tuominen J, Rauramo I, Koskenvuo M. Advance information improves user satisfaction with the levonorgestrel intrauterine system. Obstet Gynecol. 2002;99(4):608–613. 10.1016/s0029-7844(01)01764-1. 12039121

[10] Cavallaro FL, Benova L, Owolabi OO, Ali M. A systematic review of the effectiveness of counselling strategies for modern contraceptive methods: what works and what doesn’t? BMJ Sex Reprod Health. 2020;46:254–269. 10.1136/bmjsrh-2019-200377. 31826883 PMC7569400

[11] Jain AK. Examining progress and equity in information received by women using a modern method in 25 developing countries. Int Perspect Sex Reprod Health. 2016;42(3):131–140. 10.1363/42e1616. 28825904

[12] Ali MM, Cleland J, Shah IH. *Causes and Consequences of Contraceptive Discontinuation: Evidence From 60 Demographic and Health Surveys*. World Health Organization; 2012. Accessed March 8, 2023. https://apps.who.int/iris/handle/10665/75429

[13] Polis C, Hussain R, Berry A. There might be blood: a scoping review on women's responses to contraceptive-induced menstrual bleeding changes. Reprod Health. 2018;15(1):114. 10.1186/s12978-018-0561-0. 29940996 PMC6020216

[14] Littlejohn K, Kimport K. Contesting and differentially constructing uncertainty: negotiations of contraceptive use in the clinical encounter. J Health Soc Behav. 2017;58(4):442–454. 10.1177/0022146517736822. 29172767 PMC6101241

[15] Counseling for Choice. Population Services International. Accessed March 8, 2023. https://www.psi.org/C4C

[16] Holt K, Zavala I, Quintero X, Mendoza D, McCormick M, Dehlendorf C, et al. Women’s preferences for contraceptive counseling in Mexico: results from a focus group study. Reprod Health. 2018;15(1):128. 10.1186/s12978-018-0569-5. 30012157 PMC6048723

[17] Dehlendorf C, Kimport K, Levy K, Steinauer J. A qualitative analysis of approaches to contraceptive counseling. Perspect Sex Reprod Health. 2014;46(4):233–240. 10.1363/46e2114. 25040686 PMC4487742

[18] Schivone G, Glish L. Contraceptive counseling for continuation and satisfaction. Curr Opin Obstet Gynecol. 2017;29(6):443–448. 10.1097/GCO.0000000000000408. 28938374

[19] León FR, Roca S, Ríos A, Zumarán A, Feijoo AR. *One-Year Client Impacts of Quality of Care Improvements Achieved in Peru*. FRONTIERS Final Report. Population Council; 2003. 10.31899/rh4.1196

[20] Danna K, Angel A, Kuznicki J, Lemoine L, Lerma K, Kalamar A. Leveraging the client-provider interaction to address contraceptive discontinuation: a scoping review of the evidence that links them. Glob Health Sci Pract. 2021;9(4):948–963. 10.9745/GHSP-D-21-00235. 34933989 PMC8691884

[21] Population Council. *The Balanced Counseling Strategy Plus: A Toolkit for Family Planning Service Providers Working in High HIV/STI Prevalence Settings*. 3rd ed. Population Council; 2015. Accessed March 8, 2023. https://www.popcouncil.org/research/the-balanced-counseling-strategy-plus-a-toolkit-for-family-planning-service

[22] Warren CE, McClair TL, Kirk KR et al. Design, adaptation, and diffusion of an innovative tool to promote shared contraceptive decision-making: Balanced Counseling Strategy Plus [version 1; peer review: 2 approved with reservations]. Gates Open Res. 2022;6:2. 10.12688/gatesopenres.13477.1

[23] Rinehart W, Rudy S, Drennan M. GATHER guide to counseling. Popul Rep J. 1998;48:1–31. 10096107

[24] Family Health International (FHI 360). *NORMAL Counseling Tool for Menstrual Bleeding Changes*. FHI 360; 2019. Accessed March 8, 2023. https://www.fhi360.org/resource/normal-counseling-tool-menstrual-bleeding-changes

[25] Dehlendorf C, Fox E, Silverstein IA, et al. Development of the Person-Centered Contraceptive Counseling scale (PCCC), a short form of the Interpersonal Quality of Family Planning care scale. Contraception. 2021;103(5):310–315. 10.1016/j.contraception.2021.01.008. 33508252

[26] Using the Measure. Person-Centered Contraceptive Counseling Measure. Accessed March 8, 2023. https://pcccmeasure.ucsf.edu/using-measure-0

[27] Dehlendorf C, Henderson JT, Vittinghoff E, et al. Association of the quality of interpersonal care during family planning counseling with contraceptive use. Am J Obstet Gynecol. 2016;215:78.e1–e9. 10.1016/j.ajog.2016.01.173. 26827879

[28] Johnson SL, Kim YM, Church K. Towards client-centered counseling: development and testing of the WHO Decision-Making Tool. Patient Educ Couns. 2010;81(3):355–361. 10.1016/j.pec.2010.10.011. 21093194

[29] Holt K, Zavala I, Quintero X, et al. Women's preferences for contraceptive counseling in Mexico: results from a focus group study. Reprod Health. 2018;15(1):128. 10.1186/s12978-018-0569-5. 30012157 PMC6048723

[30] Teshome A, Birara M, Rominski SD. Quality of family planning counseling among women attending prenatal care at a hospital in Addis Ababa, Ethiopia. Int J Gynaecol Obstet. 2017;137(2):174–179. 10.1002/ijgo.12110. 28170078

[31] RamaRao S, Lacuesta M, Costello M, Pangolibay B, Jones H. The link between quality of care and contraceptive use. Int Fam Plan Perspect. 2003;29(2):76–83. 10.2307/3181061. 12783771

[32] Abdel-Tawab N, Roter D. The relevance of client-centered communication to family planning settings in developing countries: lessons from the Egyptian experience. Soc Sci Med. 2002;54:1357–1368. 10.1016/s0277-9536(01)00101-0. 12058852

[33] Jain AK, Ramarao S, Kim J, Costello M. Evaluation of an intervention to improve quality of care in family planning programme in the Philippines. J Biosoc Sci. 2012;44(1):27–41. 10.1017/S0021932011000460. 21933467

[34] Jain A, Aruldas K, Tobey E, Mozumdar A, Acharya R. Adding a question about method switching to the method information index is a better predictor of contraceptive continuation. Glob Health Sci Pract. 2019;7(2):289–299. 10.9745/GHSP-D-19-00028. 31249024 PMC6641810

[35] Diamond-Smith N, Warnock R, Sudhinaraset M. Interventions to improve the person-centered quality of family planning services: a narrative review. Reprod Health. 2018;15:44. 10.1186/s12978-018-0592-6. 30153846 PMC6114885

[36] Diamond-Smith N, Phillips B, Afulani P, Srivastava A, Golub G, Sudhinaraset M. Person-centred quality, provider involvement and family planning continuation in India. *Res Sq*. Preprint posted online December 15, 2020. 10.21203/rs.3.rs-124362/v1

