# Supplementary material for: Evaluating Counseling for Choice in Malawi: A Client-Centered Approach to Contraceptive Counseling
Source: Glob Health Sci Pract. 2023 Apr 28;11(2):e2200319. doi: 10.9745/GHSP-D-22-00319 (PMC10141422; doi:10.9745/GHSP-D-22-00319)
Supplement: GHSP-D-22-00319-supplement.pdf [file GHSP-D-22-00319-supplement.pdf]

Supplement to: Kalamar A, Danna K, Angel A, et al. Evaluating Counseling for Choice in Malawi: a client-centered approach to contraceptive counseling. *Glob Health Sci Pract*. 2023;11(2):e2200319. https://doi.org/10.9745/GHSP-D-22-00319

SUPPLEMENT

Study Sites

Malawi comprises 28 districts within 3 regions (Central, Northern, and Southern). The study was conducted in 8 districts across all 3 regions (Mzuzu and Nkhata Bay, districts within the Northern Region; Dwangwa, Lilongwe, Mchinji and Salima, districts within the Central Region; and Mangochi and Nsanje, districts within the Southern Region).

The sampling frame to select public sector study facilities to receive the C4C intervention consisted of a total of 57 public health centers (24in Mangochi, 12 in Nsanje, and 21 in Nkhata Bay), of which 30 were originally sampled and 25 agreed to participate and were included in the study (12 in Mangochi, 5 in Nsanje, and 8 in Nkhata Bay). These intervention facilities were then matched one-to-one with 25 comparison facilities located in the same districts.

The sampling frame to select private sector study facilities to receive the C4C intervention consisted of a total of 51 private health centers (21 in Mzuzu, 10 in Lilongwe, 10 in Dwangwa, 4 in Nkhatabay, 4 in Mchinji, and 2 in Salima), of which 30 were originally sampled and 20 agreed to participate in the C4C intervention and were included in the study (5 in Mzuzu, 5 in Lilongwe,5 in Dwangwa, 2 in Nkhatabay, 2 in Mchinji, and 1 in Salima). As for the public sector, private intervention facilities were matched one-to-one with 20 comparison facilities located in the same districts.

While the original intention was to select private facilities from the same districts from which public facilities were selected, the lack of eligible facilities in these districts required expanding the number of districts included for the private sector study sites.

Sample Size Considerations

Analyses were conducted using data that was collected as part of a prospective cohort study with follow-up scheduled at 6 months post clinic visit. For this analysis we have included only the baseline data from this study (i.e., immediate pre- and post-counseling surveys). Because the study was powered based on a 6-month outcome, no *a priori* sample size calculations are available for the immediate post-counseling variables presented in these analyses. Similarly, no primary post-counseling outcome variable was specified *a priori*. Below we provide the original sample size calculations as context.

Target sample size for the prospective cohort study in which these data are nested was based on the ability to detect a 15% difference in all method continuation between the intervention and control groups at 12 months, using a baseline contraception discontinuation prevalence of 37% in Malawi, according to the 2015-16 MDHS,[21] with a 95% confidence interval (α=0.05), and 80% power (β=0.8). Using these inputs and assumptions, the minimum sample size in each study group - intervention-private, intervention-public, control-private, and control-public – was calculated to be 185 women. To account for intraclass correlations (ICC), a design effect of 1.2 was assumed; loss to follow-up, given the length of the study and the use of mobile phones, was assumed to be 20% at 6 months. Taking these adjustments into account, the intended sample size at baseline was 267 women per group, for a total of 1,068 women to be enrolled in the study at baseline. Women were then recruited from the selected intervention and control facilities in numbers proportional to client flow

so that the total sample size across all facilities reached the intended minimum required sample size for each group (Table 1).

Table 1. Participant Recruitment by Intervention arm and Facility Type

	Control (N=578)			Intervention (N=601)		
	Facility #	No	%	Facility #	No	%
**Public Facility**	102	9	1.6	104	5	0.8
	107	5	0.9	106	6	1.0
	111	1	0.2	108	6	1.0
	115	9	1.6	110	3	0.5
	120	7	1.2	113	2	0.3
	201	6	1.0	114	2	0.3
	202	10	1.7	118	3	0.5
	203	13	2.3	121	4	0.7
	204	18	3.1	206	15	2.5
	205	9	1.6	207	2	0.3
	209	14	2.4	208	26	4.3
	210	17	2.9	211	20	3.3
	213	23	4.0	212	10	1.7
	214	17	2.9	215	2	0.3
	218	1	0.2	216	32	5.3
	220	23	4.0	217	16	2.7
	224	12	2.1	219	9	1.5
	301	17	2.9	221	9	1.5
	302	23	4.0	222	5	0.8
	303	8	1.4	223	27	4.5
	305	23	4.0	304	19	3.2
				306	10	1.7
				307	23	3.8
				308	11	1.8
	**Total**	265	45.8	**Total**	267	44.4
**Private Facility**	101	15	2.6	117	11	1.8
	105	8	1.4	122	50	8.3
	402	12	2.1	404	2	0.3
	403	49	8.5	405	17	2.8
	407	7	1.2	406	20	3.3
	408	15	2.6	410	2	0.3
	409	14	2.4	411	3	0.5
	501	8	1.4	502	15	2.5
	503	50	8.7	504	13	2.2

	602	33	5.7	601	9	1.5
	603	13	2.3	605	1	0.2
	604	11	1.9	608	23	3.8
	606	14	2.4	609	26	4.3
	607	9	1.6	610	21	3.5
	701	5	0.9	702	29	4.8
	703	21	3.6	704	53	8.8
	802	8	1.4	801	1	0.2
	804	17	2.9	803	13	2.2
	902	4	0.7	901	25	4.2
	**Total**	313	54.2	**Total**	334	55.6

Midway through baseline recruitment, loss to follow-up (by virtue of not having a mobile phone for the six-month follow-up survey) was higher than anticipated and so the study team made the decision to oversample at baseline from the private sector but because mobile phone ownership was so low among clients in the public sector, the decision was made to not likewise oversample in the public sector. In total, we increased the original sample size by 10% to enroll a total of 1,179 female participants, 54.9% (647/1,179) of whom were enrolled from private facilities.

Quality of Care Domains

In the design of this study, authors considered several validated measures of person-centered care (the PCCC) in addition to questions designed to measure the impact of the C4C approach across the three quality of care domains that are most relevant to contraceptive counseling. The validated PCCC measures (both individual items and summative score) are presented together in their entirety in the manuscript while all other measures of quality related to informed decision making and side effects counseling are presented in Tables 2 and 4, respectively. However, some PCCC measures also relate to these domains. The table below explains how authors envision how each variable maps to an individual quality of care domain.

Supplement to: Kalamar A, Danna K, Angel A, et al. Evaluating Counseling for Choice in Malawi: a client-centered approach to contraceptive counseling. *Glob Health Sci Pract*. 2023;11(2):e2200319. https://doi.org/10.9745/GHSP-D-22-00319

Table 2. Survey Questions and Quality of Care Domains

Question	Responses	Quality of Care Domain	Origin
From 1 to 5, with 1 being very poor and 5 being very excellent, how would you rate the counseling you received from the provider?	1- Poor<br>2-Fair<br>3- Good<br>4- Very good<br>5- Excellent		
Who talked more during your counseling session?	00- Provider talked most of the time<br>01- The provider and I talked the same amount<br>02- I talked most of the time<br>03- Don't Know	Information exchange	
Did the provider ask about your past experience with contraception?	00- No<br>01- Yes<br>02- Don't know	Information exchange	
Did the provider address all concerns you had about using contraception?	00- No<br>01- Yes<br>02- Don't know	Information exchange	
Do you trust that the provider will keep what you discussed a secret?	00- No<br>01- Yes<br>02- Don't know	Interpersonal Relations	
Think about your visit. How do you think your provider did? Please tell me on a scale from 1 to 5, with 1 being poor and 5 being excellent: Respecting me as a person	1- Poor<br>2-Fair<br>3- Good<br>4- Very good<br>5- Excellent	Interpersonal Relations	PCCC
Think about your visit. How do you think your provider did? Please tell me on a scale from 1 to 5, with 1 being poor and 5 being excellent: Letting me say what mattered to me about my birth control method	1- Poor<br>2-Fair<br>3- Good<br>4- Very good<br>5- Excellent	Information exchange	PCCC
Think about your visit. How do you think your provider did? Please tell me on a scale from 1 to 5, with 1 being poor and 5 being excellent: Taking my preferences about my birth control seriously	1- Poor<br>2-Fair<br>3- Good<br>4- Very good<br>5- Excellent	Interpersonal relations	PCCC
Think about your visit. How do you think your provider did? Please tell me on a scale from 1 to 5, with 1 being poor and 5 being excellent: Giving me enough information to make the best decision about my birth control method	1- Poor<br>2-Fair<br>3- Good<br>4- Very good<br>5- Excellent	Information exchange	PCCC
On a scale of 1 to 5, with 1 being not at all and 5 being very, how confident do you feel that you understand how to use your method correctly?	1- Not at all confident<br>2-<br>3-<br>4-<br>5- Very confident	Information exchange	
Did the provider discuss with you the type of side effects you can expect from your chosen method of contraception?	00- No<br>01- Yes	Anticipatory Side Effects	
Did the provider help you make a plan for how to remember to use your method correctly?	00- No<br>01- Yes	Information Exchange	
What will you do if you experience side effects?	00- Wait to see if they go away<br>01- Keep using anyways<br>02- Stop using the method immediately<br>03- Try a different method<br>04- Return to the clinic/Talk to a provider<br>05- Talk to an IPC worker<br>06- Talk to someone else (specify):<br>07- Other (specify):	Anticipatory Side Effects	
Did the provider help you make a plan for how to manage side effects?	00- No<br>01- Yes	Anticipatory Side Effects	

Supplement to: Kalamar A, Danna K, Angel A, et al. Evaluating Counseling for Choice in Malawi: a client-centered approach to contraceptive counseling. *Glob Health Sci Pract*. 2023;11(2):e2200319. https://doi.org/10.9745/GHSP-D-22-00319

Data Collection Procedures

All data collection instruments were translated into Chichewa and back-translated for accuracy during study training by the study team. All research assistants were trained in public health, had quantitative data collection experience, and spoke fluent Chichewa.

Ethical Procedures

Oral consent was obtained due to the sensitive nature of the data around family planning and healthcare utilization. All research assistants signed and dated the consent form to indicate receipt of verbal consent and a copy of the consent form was provided to the participants. The District Health Management Team and the individual in charge at each facility gave their permission for data collection.

Description of Counseling for Choice Approach

Foundational to the C4C approach are three contraceptive counseling tenets: support the client to make an informed decision through clear and relevant information provision, provide high quality client-centered interpersonal care, and create a dialogue with clients about side effects, including what to expect and how to manage them. The C4C approach in Malawi has three key components: tools for training and supervision of providers, a three-day training for providers, and the Choice Book job aid for providers to use during counseling.

The program implementing this study in Malawi trained all study providers together in the same 3-day training. The training that providers receive is designed to give them multiple tools and techniques to improve the counseling interaction. For example, the GATHER approach is a signal to providers to launch a two-way conversation that begins by 'G'reeting the client and 'A'sking them about their concerns.[i] The conversation that follows is given a flexible structure that is driven by clients' stated needs. By creating a dialogue about what matters to the client, rather than using the counseling session as a didactic lecture to impart the provider perspective (and potential bias), C4C aims to make clients feel more confident that they have been heard and can make a decision about the best method for them.

Figure S1. Counseling matrix in the C4C Choice Book

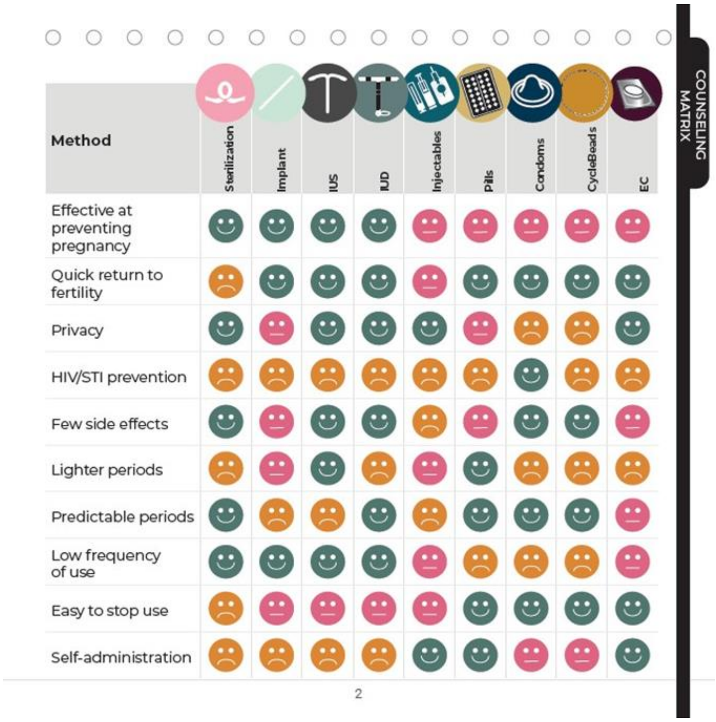

The Choice Book for providers is a job aid that includes both provider-facing and client-facing tools and references. A C4C counseling session begins by comparing the attributes and potential benefits that might be of interest to the client. To enable informed decision-making, the counseling matrix (Figure 1) included in the Choice Book is the keystone tool which orients clients to all the characteristics of family planning that might be important to them-allowing them to state their preferences and direct the

conversation from the start. From that initial point, subsequent pages of the Choice Book delve into these specific method attributes so that clients can see how each method compares relative to their priorities. Understanding the client's preferences, the provider then uses this framework to help the client understand her different options, for example if a client responds that privacy is of greatest importance, the provider can use the specific page on privacy (Figure 2) to help compare method options relative to this benefit. The intention is for the client to be matched with a method that will suit her and avoid overload of information she might otherwise face through a standard counseling approach.

Figure 2. Page comparing privacy attributes of different methods

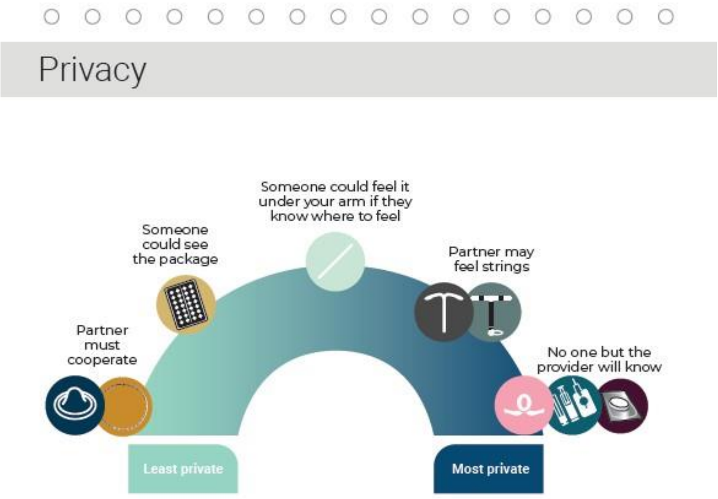

Counseling approaches often lack an adequate focus on how to counsel clients about possible side effects.[13] The C4C approach contains several tools to prepare the client for possible side effects and how to manage them. The NORMAL tool and pneumonic device help to remind clients that bleeding changes are normal and safe, expected with certain methods, reversible, and may present potential lifestyle opportunities. This tool helps the provider to explain to a client how she might expect her bleeding to change with her method options.

When a woman has chosen a method that appeals to her, the Choice Book signals the provider to assess her medical eligibility using tools provided within the Book and ensure that her chosen method is suitable. It also signals the provider to discuss key continuity of care messages, such as 'the 3Ws' (what to do, what to expect, when to come back) and to help the client make a plan to manage common side effects. The Choice Book also includes reference tools for the WHO 2015 medical eligibility criteria (MEC), post-partum and post-abortion eligibility, and a job aid that tells providers when to use the pregnancy checklist to rule out pregnancy, versus when a pregnancy test is recommended. Through the C4C approach, providers learn to use the Choice Book during a counseling training in which they have plenty of opportunity for practice and role-play.

[i] Rinehart W, Rudy S, Drennan M. GATHER guide to counseling. Popul Rep J. 1998;48:1-31.
